# Epidemiology, Clinical Manifestations, and Outcomes of *Streptococcus suis* Infection in Humans

**DOI:** 10.3201/eid2007.131594

**Published:** 2014-07

**Authors:** Vu Thi Lan Huong, Ngo Ha, Nguyen Tien Huy, Peter Horby, Ho Dang Trung Nghia, Vu Dinh Thiem, Xiaotong Zhu, Ngo Thi Hoa, Tran Tinh Hien, Javier Zamora, Constance Schultsz, Heiman Frank Louis Wertheim, Kenji Hirayama

**Affiliations:** Oxford University Clinical Research Unit, Hanoi, Vietnam (V.T.L. Huong, P. Horby, H.F.L. Wertheim);; University of Oxford, Oxford, UK (V.T.L. Huong, P. Horby, H.F.L. Wertheim, N.T. Hoa);; Nagasaki University, Nagasaki, Japan (N. Ha, N.T. Huy, X. Zhu, K. Hirayama);; Oxford University Clinical Research Unit, Ho Chi Minh City, Vietnam (H.D.T. Nghia, N.T. Hoa, T.T. Hien, C. Schultsz);; National Institute of Hygiene and Epidemiology, Hanoi (V.D. Thiem);; Ramón y Cajal Hospital, Madrid, Spain (J. Zamora);; CIBER Epidemiologia y Salud Publica, Madrid (J. Zamora);; Pham Ngoc Thach University of Medicine, Ho Chi Minh City (H.D.T. Nghia);; University of Amsterdam, Amsterdam, the Netherlands (C. Schultsz)

**Keywords:** *Streptococcus suis*, bacterial meningitis, systematic review, meta-analysis, zoonoses, bacteria, humans

## Abstract

*Streptococcus suis,* a bacterium that affects pigs, is a neglected pathogen that causes systemic disease in humans. We conducted a systematic review and meta-analysis to summarize global estimates of the epidemiology, clinical characteristics, and outcomes of this zoonosis. We searched main literature databases for all studies through December 2012 using the search term “streptococcus suis.” The prevalence of *S. suis* infection is highest in Asia; the primary risk factors are occupational exposure and eating of contaminated food. The pooled proportions of case-patients with pig-related occupations and history of eating high-risk food were 38.1% and 37.3%, respectively. The main clinical syndrome was meningitis (pooled rate 68.0%), followed by sepsis, arthritis, endocarditis, and endophthalmitis. The pooled case-fatality rate was 12.8%. Sequelae included hearing loss (39.1%) and vestibular dysfunction (22.7%). Our analysis identified gaps in the literature, particularly in assessing risk factors and sequelae of this infection.

## Abstract 2

Infection occurs mainly in Asia; occupational and food exposures are the primary risk factors.

*Streptococcus suis* is a neglected zoonotic pathogen that has caused large outbreaks of sepsis in China (*1*,*2*) and has been identified as the most common and the third leading cause of bacterial meningitis in adults in Vietnam and Hong Kong, respectively (*3*–*5*). *S. suis* infection is acquired from pigs, either during slaughtering or by handling and eating undercooked pork products. It is potentially preventable (*3*,*6*). Epidemiology of the infection differs between Western and Asian regions (*7*), and the role of high-risk eating habits (i.e., ingesting raw or undercooked pig parts, including pig blood, organs, and meat) in some Asian communities recently has been recognized (*6*,*8*,*9*). Rates of *S. suis* infection are low in the general populations of Europe and North America, and cases are concentrated among occupationally exposed groups, including abattoir workers, butchers, and pig breeders (*10*,*11*).

In a 2009 review, ≈700 *S. suis* infections were reported worldwide by 2009, mostly from China and Vietnam (*12*). Clinical characteristics of this infection have been reviewed (*12*,*13*) and include meningitis, sepsis, endocarditis, arthritis, hearing loss, and skin lesions. Treatment of *S. suis* infection requires ≈2 weeks of intravenous antimicrobial drugs (*12*). The death rate varies, and deafness is a common sequela in survivors.

Although substantial new data on the incidence, clinical and microbiological characteristics, and risk factors for *S. suis* infection have accumulated during recent years, the prevalence of this infection has not measurably decreased. We conducted a systematic review and meta-analysis to update the evidence and summarize the estimates of epidemiologic and clinical parameters to support practitioners’ and policy makers’ efforts to prevent and control this infection.

## Methods

We conducted the review in accordance with recommendations of the PRISMA statement (*14*). The protocol for this review has been registered at PROSPERO International prospective register of systematic reviews (no. CRD42011001742).

### Search Strategy and Selection Criteria

**Figure 1:**
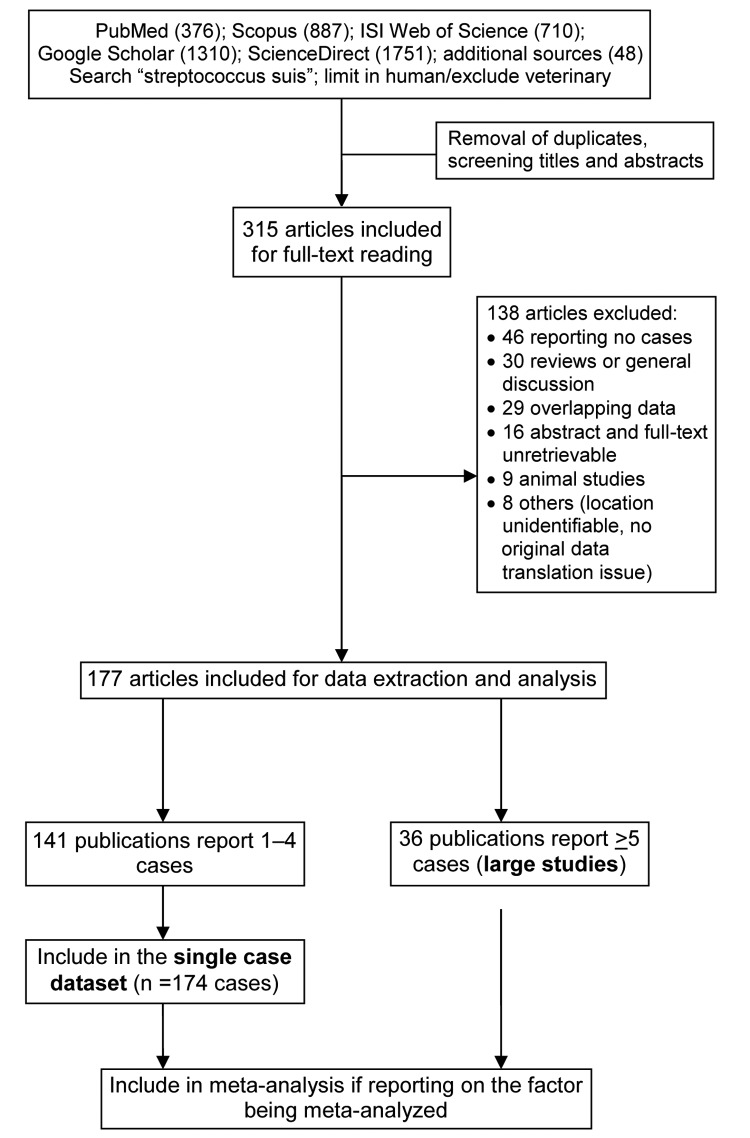
Flow diagram of the search and review process for this review of *Streptococcus suis* infection.

We systematically and inclusively searched 5 main electronic databases (PubMed, Scopus, ISI Web of Science, Science Direct, and Google Scholar) for all studies published until the end of December 2012 (Figure 1). The following search term was used as a text word in each database, as follows: PubMed—“streptococcus suis” in all fields, limited to humans; Scopus—“streptococcus suis” in all fields, excluding veterinary medicine articles; ISI Web of Science—“streptococcus suis” in topic with exclusion of veterinary science areas; Science Direct—“streptococcus suis” in all fields, with articles in veterinary medicine journals excluded; and Google Scholar—“allintitle: ‘streptococcus suis’”

We also searched using the same search term “streptococcus suis” in other databases, including Virtual Health Library, SIGLE, WHOLIS, LILACS, IMSEAR-HELLIS, and China Academic Journals Full-text Database and checked the reference lists of retrieved articles. We did not restrict the types of studies and publication languages, and non-English papers were translated for review. Publications were excluded if they did not report any human cases of *S. suis* infection, reported data that overlapped with already included articles and provided no additional information, reported cases without information indicating the location of the patients, or reported data that could not be reliably extracted.

### Data Extraction

Two reviewers (N.H. and V.T.L.H.) independently screened the titles and abstracts, and examined the full-text publications by using identical selection criteria and data abstraction forms. The results of data extraction showed a high degree of agreement between the reviewers (κ>0.90 for the main variables). Any disagreements were resolved by discussion and consensus between the reviewers and other authors (N.T. Huy, H.W., P.H., K.H.). We emailed the original authors of the articles that contained ambiguous data (1 email attempt per author) for clarification, and the ambiguous data were excluded from analyses if we did not receive a response.

Data extracted included year of publication, year of data collection, study design, data collection approach, country of origin, hospital where the patients were recruited, patient characteristics, clinical manifestations, methods of diagnosis, clinical and laboratory parameters, outcomes, and histories.

### Analyses

We described the relevant epidemiologic and clinical factors using count for number of cases, proportions with 95% CIs for categorical factors (sex, occupation, exposure, history), and mean with SD for continuous factors (age, duration, and laboratory parameters). Event rates are presented as proportions with 95% CIs for signs, symptoms, and outcomes. We defined an event rate as the ratio of number of events to the number of all patients assessed in each study.

We pooled all single cases from the publications that reported <5 cases into 1 dataset and produced summary outputs, which were then meta-analyzed with other large studies (reporting >5 cases). We report the values of reviewed factors in 3 sets: summary values from the single-case dataset, median values (range) of the large studies, and pooled values from the meta-analysis as appropriate.

Meta-analysis was conducted by using Comprehensive Meta-analysis software version 2 (Biostat, Englewood, NJ, USA; http://www.Meta-Analysis.com) when >2 studies reported the reviewed factor. We tested heterogeneity using the Q statistic and I^2^ test (*15*). Pooled values and 95% CIs were generated from a fixed-effects model or from a random-effects model, and each was study weighted by the inverse of that study’s variance. We used the fix-effects model when heterogeneity was not significant and a random-effects model when heterogeneity was evident (*16*). Median (range) was converted to mean (SD) by using proposed formulas (*17*), and interquartile ranges to SDs and subgroup values to total values by Cochrane suggested methods (*18*).

We assessed publication bias using funnel plots and the Egger’s regression test (if >10 studies were included in the meta-analysis). If publication bias was found, the Duvall and Tweedie trim and fill method was performed to add possible missing studies to improve the symmetry and calculate the adjusted pooled values (*19*). We used subgroup analyses (when >10 studies were included) and bivariate meta-regression (when >20 studies were included) to examine the effect of study-level variables, including year of publication (2005 and earlier vs. after 2005 [because the Sichuan outbreak occurred in 2005]), study design (case series, outbreak investigation, cross-sectional), location (China mainland, Hong Kong, Thailand, Vietnam, and others), data collection (retrospective vs. prospective) and meningitis rate (<50%, 50%–90%, and >90%) on the main outcomes. The general linear model was used for meta-regression, with adjustment for multiple comparisons by using the Bonferroni method and weighting by study sample size.

## Results

### Systematic Review

We used 177 publications that met inclusion and exclusion criteria for data extraction and final analyses (Figure 1; Technical Appendix Table 1). The studies were diverse in terms of design, data collection, and reporting approaches. We identified 20 case series (8 from South-East Asia region, 8 from the Western Pacific region, and 4 from Europe) and 21 cross-sectional studies (9 SouthEast Asia, 8 Western Pacific, and 4 Europe). Five articles about 3 outbreaks (in Sichuan and Jiangsu, China; and Phayao, Thailand) were classified as outbreak investigation reports. The only prospective case–control study was conducted in Vietnam (Table 1).

**Table 1:** Characteristics of 177 articles in a systematic review of *Streptococcus suis* infection

Characteristic	Articles, no. (%)	Cases reported, no. (%)*
Geographic region†		
Europe	98 (55)	168 (11)
Western Pacific	47 (27)	836 (53)
SouthEast Asia	24 (14)	572 (36)
Americas	8 (5)	8 (0.5)
Type of study design		
Case report	130 (73)	151 (7)
Case series	20 (11)	511 (25)
Cross-sectional	21 (12)	761 (37)
Outbreak investigation	5 (3)‡	532 (26)
Case–control	1 (1)	101 (5)
Data collection approach		
Retrospective	159 (90)	1299 (63)
Prospective	15 (9)	697 (34)
Both§	3 (1)	60 (3)
Language of publication¶		
English	130 (74)	1947 (95)
Spanish	13 (7)	15 (1)
French	12 (7)	13 (1)
Other#	22 (12)	81 (4)
Year of publication		
1968–1980	13 (7.5)	18 (1)
1981–1990	27 (15)	95 (5)
1991–2000	32 (18)	119 (6)
2001–2005	28 (16)	115 (6)
2006–2010	55 (31)	1052 (51)
2011–2012	22 (12.5)	659 (32)

*Case duplicates were removed in the counts for the geographic region subheading (totaling 1,584 cases, no duplicates). Duplicates were not removed in the counts for other subheadings (totaling 2,056 cases, with duplicates). †Geographic regions as defined by the World Health Organization. ‡Includes 3 articles reporting about the patients in the Sichuan outbreak in China; each was included for analysis of different factors. §Included in the prospective groups in subsequent analyses. ¶Almost all large studies were published in English. Most reports in languages other than English were case reports. #German (7 articles); Dutch (4); Czech, Italian, and Japanese (2 each); Chinese, Polish, Serbian, Swedish, and Thai (1 each).

### Epidemiology

**Figure 2:**
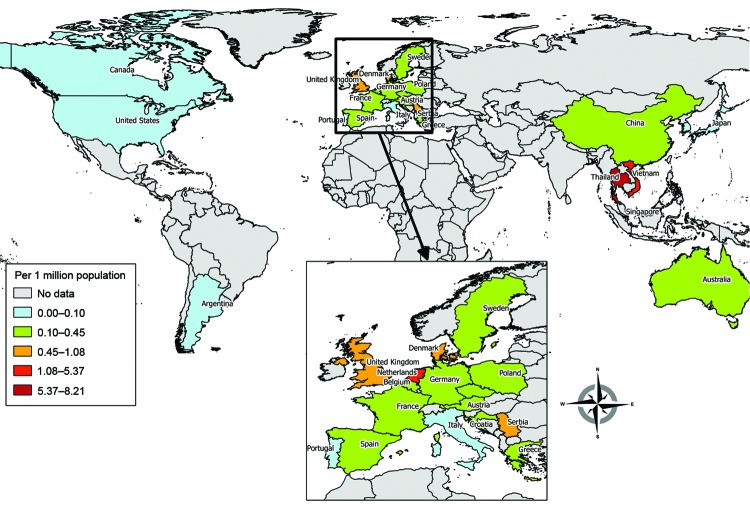
Global cumulative prevalence of *Streptococcus suis* infection through 2012.

By the end of 2012, a total of 1,584 cases had been reported in the literature (including 189 probable cases identified in 3 outbreaks), mainly from Thailand (36%), Vietnam (30%), and China (22%). More than half (53%) were in the Western Pacific region; 36% were in the South East Asia region, 10.5% in the European region, and 0.5% in the Americas. The highest cumulative prevalence rate was in Thailand (8.21 cases/million population), followed by Vietnam (5.40) and the Netherlands (2.52) (country population data for 2008–2012 by World Bank [*20*]) (Figure 2).

The pooled mean age of the patients was 51.4 years, and 76.6% were men (Table 2). All case-patients were adults, except 1 female infant reported in Thailand (*21*). The pooled proportion of case-patients with occupational exposure was 38.1% (95% CI 24.4%–53.9%); this proportion was higher for industrialized countries than for other countries (83.8% [95% CI 73.4%–90.7%] for the United Kingdom, Netherlands, and Japan together). Recent contact with pigs or pork was reported for 15.5% of single cases but for 33.9% (95% CI 21.1%–49.5%) in the meta-analysis. History of eating meals containing pork was reported mainly in Asia (Thailand and Vietnam); the pooled estimate was 37.3% (95% CI 20.2%–58.3%). For Thailand only, the proportion was 55.8% (95% CI 33.7%–75.9%). In other countries, only 1 patient in France was reported eating artisanal dry sausage (*22*), and 1 patient in the United States ate raw pork while traveling in the Philippines (*23*) before the infection.

**Table 2:** Epidemiologic factors of patients with *Streptococcus suis* infection included in a systematic review

Variable	Single-case dataset, %*	Large studies, median (range), %	Meta-analysis, pooled mean (95% CI), %†	No. studies meta-analyzed, %‡
Mean age, y, n = 156	49.4	50.5 (37.0–61.2)	51.4 (49.5–53.2)	25
Male sex, n = 155	83.2	77.5 (37.5–100)	76.6 (72.2–80.6)	26
Pig-related occupation	58.6	25.0 (0–100)	38.1 (24.4–53.9)	21
Contact with pig/pork	15.5	33.3 (2.4–100)	33.9 (21.1–49.5)	14
Eating of high-risk food	4.0	53.3 (5.9–88.7)	37.3 (20.2–58.3)	9
Skin injury	19.5	16.0 (9.5–71.4)	25.1 (15.1–38.7)	8
Drinking of alcohol	8.6	23.0 (4.8–83.9)	29.7 (17.2–46.3)	13
Concurrent diabetes§	2.9	7.2 (3.2–25.0)	8.0 (4.6–13.7)	9

*N = 174 unless otherwise indicated. †Random-effects model unless otherwise specified. ‡Include the single-case dataset and the large studies (Technical Appendix Table 2). §Other less common underlying conditions are listed in Technical Appendix Table 3.

Skin injury was shown for one fourth of case-patients, and alcohol use was evident in approximately one third of case-patients. However, a case–control study in Vietnam did not identify alcohol use as an independent risk factor after adjustment for other risk factors and confounders (*6*). The most commonly reported preexisting condition was diabetes. Other conditions included underlying heart disease, hypertension, cirrhosis, and cancer (Technical Appendix Table 3). Smoking was mentioned in 5.2% of patients in the single-case dataset.

### Microbiological Diagnosis

Blood and/or cerebrospinal fluid culture were the most common reported diagnostic methods among the case reports (Technical Appendix Table 4). Molecular diagnosis was more common in the large studies (11 studies) than in case reports. The most prevalent strain was serotype 2 (86.5%), followed by serotype 14 (2.3%), and serotype 1 (0.6%) of all 1,156 patients with serotype information mentioned in the articles. Serotypes 4, 5, 16, and 24 also were reported (1 patient per serotype).

Misdiagnosis of *S. suis* infection was not uncommon, either by conventional biochemical tests or commercial identification systems. The bacteria were often reported as viridans streptococci in initial cultures. In fact, up to 70% of all viridans streptococci cases in Thailand were confirmed as *S. suis* infections in the follow-up investigations (*24*). A total of 62.5% of *S. suis*–infected patients in another study in Thailand (*25*) and 20% in a study inthe Netherlands (*10*) were initially reported to be infected with viridans streptococci. Misidentification of the infectious agent as *S. bovis* (2 patients), *S. pneumoniae* (1 patient), and *S. faecalis* (1 patient) also was reported in the Netherlands series. Tsai et al. (*26*) showed large variations between the 2 commercial systems (Phoenix Identification System, Beckon Dickinson, Sparks, MD, USA; and Vitek II GPI Card, bioMérieux Vitek, Hazelwood, MO, USA), and misidentification of *S. suis* as *S. acidominimus* was common when the Phoenix system was used.

*S. suis* is mostly sensitive to penicillin; resistance was reported in only 2 patients (*21*,*27*). After cessation of antimicrobial drug treatment, infection relapsed in a small proportion of patients, including those for whom the organism tested highly sensitive to penicillin (*28*,*29*). The pooled relapse rate was 4.4% (Table 3).

**Table 3:** Main clinical and laboratory parameters at admission of the patients with *Streptococcussuis* infection in a systematic review

Variable	Single-case dataset	Large studies, median value (range)	Meta-analysis, pooled mean (95% CI)†	No. studies meta-analyzed‡
Clinical syndrome, %§				
Meningitis	69.5	64.5 (30.2–100)	68.0 (58.9–75.8)	26
Sepsis¶	19.5	23.8 (11.8–39.4)	25.0 (20.5–30.2)	12
Arthritis	2.87	16.7 (1.5–50.0)	12.9 (6.0–25.6)	12
Endocarditis	8.6	14.3 (1.9–39.0)	12.4 (6.7–21.9)	10
Endophthamiltis	2.9	4.5(1.5–28.6)	4.6 (2.8–7.4)#	9
Spondylodiscitis	4.6	1.9 (1.5-2.4)	3.7 (2.1-6.6)	4
Toxic shock syndrome	2.9**	37.7 (28.9–64.0)	25.7 (9.8-52.6)	4††
Mean duration, d				
Onset to admission, n = 90	7.3	3.5 (2.0–11.4)	4.1 (2.7–5.4)	7
Hospitalization, n = 68	20.5	17.4 (13.0–19.2)	17.2 (15.6–18.9)#	5
Symptoms, %				
Meningeal sign‡‡	49.4	66.7 (12.5–95.1)	67.1 (54.9–77.4)	18
Skin rash	10.9	12.5 (0–52.0)	15.4 (8.6–25.9)	10
Shock	8.6	11.8 (1.3–64.0)	11.9 (6.3–21.5)	12
Respiratory failure	5.2	20.0 (8.3–35.8)	16.7 (8.6–29.9)	6
Acute renal failure	5.2	8.3 (1.3–28.0)	7.1 (2.2–20.5)	5
Disseminated intravascular coagulation	10.3	6.0 (2.4–57.1)	10.3 (5.4–18.8)	9
Relapse	2.9	7.3 (2.9–8.3)	4.4 (2.4–7.8)#	5
Laboratory values (mean)§§				
Leukocytes, 10^9^ cells/L, n = 98	17.4	15.1 (13.9–18.2)	15.8 (14.6–16.9)	9
Hemoglobin, g/L, n = 22	106.7	–	–	–
Platelets, 10^9^/L, n = 41	121.0	182.4 (115–241.5)	164.9 (132.9–197)	7
Blood glucose, mg/dL, n = 32	147.8	–	–	–
C-reactive protein, mg/L, n = 36	349.7	–	–	–
Cerebrospinal fluid				
Leukocytes, cells/mm^3^, n = 88	3,166	2029 (450–3253)	2330 (1721–2939)#	7
Protein, g/L, n = 74	3.20	2.35 (1.7–4.18)	2.45 (1.91–2.99)	7
Glucose, mg/dL, n = 70	20.9	8.60 (1.7–25.6)	12.6 (3.5–21.7)	6

*N = 174 unless otherwise indicated. –, not applicable because no large study reported these data. †Random-effects model unless otherwise indicated. ‡Includes the single-case dataset and the large studies (Technical Appendix Table 2. §Other less common syndromes included peritonitis, myositis, pneumonia, sacroiliitis, abdominal aortic aneurysm, hemorrhagic labyrinthitis, gastroenteritis, vertebral osteomyelitis, lymphadenopathy, cellulitis, and vertigo. ¶Case-patients with toxic shock syndrome in China and in Thailand not included in this sepsis category. #Mixed-effects model. **Counted if the author described the case as toxic shock syndrome. ††Include 3 large studies reporting toxic shock syndrome, including 2 outbreaks in China (*2,30*) and 1 prospective study in Thailand (*24*). ‡‡Mainly reported with neck stiffness. §§Reference values may differ among laboratories. Commonly used reference values for presented laboratory blood tests are as follows: leukocytes 4.0–10 × 10^9^ cells/L; hemoglobin 140–170 g/L (for male patients) and 120–160 g/L (for female patients); platelets 150–350 × 10^9^/L; blood glucose (fasting) 70–100 mg/dL; C-reactive protein 0–8.0 mg/L. Reference ranges for cerebrospinal fluid are as follows: leucocytes 0–5 cells/mm^3^; protein 0.15–0.60 g/L; glucose 40–80 mg/dL. (Source: http://im2014.acponline.org/for-meeting-attendees/normal-lab-values-reference-table/)

### Clinical Syndromes

Meningitis was the most common clinical syndrome (pooled rate 68.0% [95% CI 58.9%–75.8%]), followed by sepsis (25.0% [95% CI 20.5%–30.2%]), arthritis (12.9% [95% CI 6.0%–25.6%]), endocarditis (12.4% [95% CI 6.7%–21.9%]), and endophthalmitis (4.6% [95% CI 2.8%–7.4%]) (Table 3). Toxic shock syndrome also was reported as a distinct severe clinical feature at high rates in 2 outbreaks in China (64.0% and 28.9% of patients) (*2*,*30*) and in Thailand (37.7%) (*24*) but at a rate of only 2.9% among the case reports.

**Figure 3:**
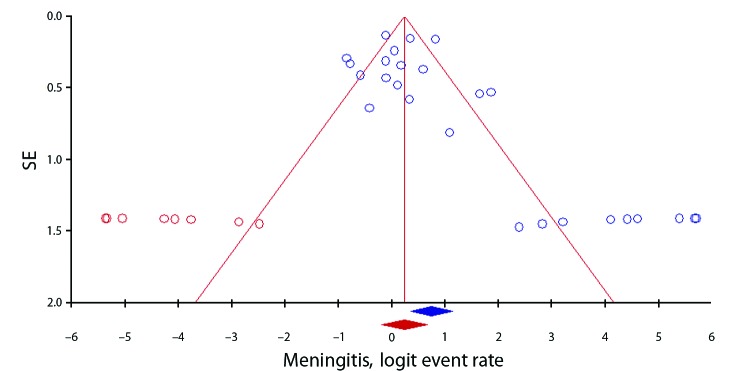
Funnel plot showing evidence of publication bias among 26 studies in a meta-analysis of meningitis rates in *Streptococcus suis* infection. Each blue circle represents each study in the meta-analysis, forming an asymmetric funnel plot with a pooled log event rate (blue rhombus). Eight missing studies (red circles) added in the left side through the trim and fill method to make the plot more symmetric and gave an adjusted log event rate (red rhombus), which was lower than the original one.

We found evidence of publication bias in the meta-analysis of meningitis rates (Figure 3) (significant Egger’s test result). The adjusted rate, based on the Duvall and Tweedie trim and fill method, was 56.0% (95% CI 45.2%–66.2%). Our meta-regression analysis showed that meningitis rate was significantly associated with country of publication, study design, and data collection approach (Technical Appendix Table 5), although only country of publication remained significant in a multivariable model. All patients in the 4 studies conducted in Vietnam had meningitis. When we excluded these studies, the pooled rate of meningitis was reduced to 59.4% (95% CI 51.1%–67.1%), and the adjusted value after we used the trim and fill method was 54.8% (95% CI 46.0%–63.4%). In contrast, if we excluded the 2 outbreak investigations in China, because sepsis dominated these outbreaks, the pooled meningitis rate increased slightly to 72.2% (95% CI 62.4%–80.2%).

### Case-Fatality Rates

**Figure 4:**
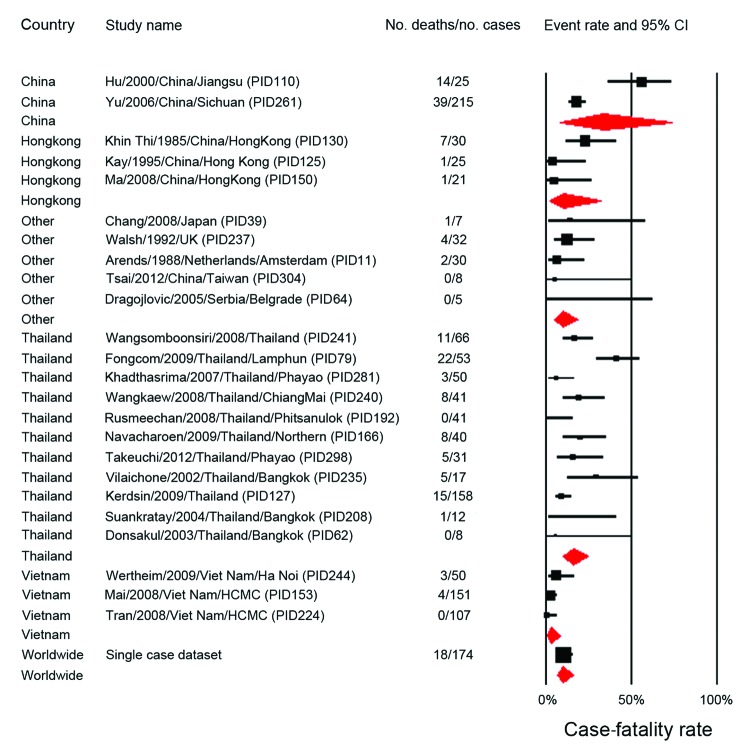
Forest plot of subgroup meta-analysis (random effects) for the case-fatality rates by country reported in the 25 studies included in a review of *Streptococcus suis* infection. For each study, the event rate of the death outcome and 95% CI are presented, with size proportional to study weight. The red rhombus indicates the pooled event rate for each country group.

**Figure 5:**
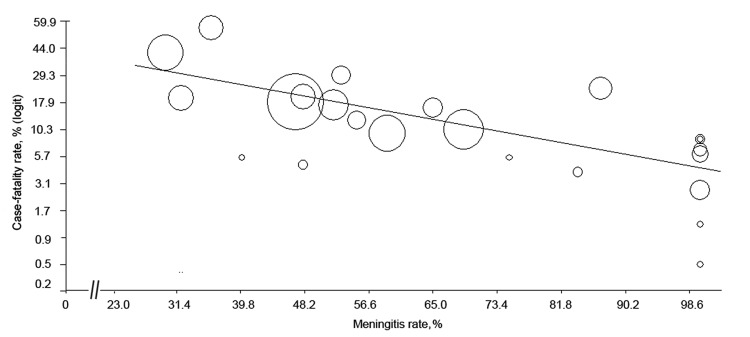
Meta-regression scatter plot showing the correlation between case-fatality rate and meningitis rate in a review of *Streptococcus suis* infection. The logit event rate was calculated for case-fatality rate as follows: logit event rate = ln[event rate/(1 − event rate)]. Each circle represents a study in the meta-analysis, and the size of the circle is proportional to study weighting. Studies with higher meningitis rates tended to report lower death rates.

The pooled case-fatality rate (CFR) for *S. suis*–infected patients was 12.8% (95% CI 9.0%–18.0%) (Table 4). This rate varied by country; reported rates were lowest in Vietnam (Figure 4). However, country of publication was not significant in the bivariate meta-regression after adjustment for multiple comparisons (Technical Appendix Table 5). Instead, only meningitis rates remained significant in explaining between-study variations in CFR. Meningitis rates correlated negatively with CFRs among the included studies (Figure 5). Studies with meningitis rates <50% had significantly higher CFRs than did studies with meningitis rates >90% (mean CFR difference 20.3%, p = 0.001). The pooled CFR was 4.0% (95% CI 2.2%–7.0%), estimated for the studies in which all patients had meningitis (*3*,*4*,*9*,*10*,*31*–*33*), whereas the pooled rate for the other studies was 17.1% (95% CI 12.3%–23.4%). CFRs were higher for outbreaks than for nonoutbreaks (21.6% [95% CI 6.4%–52.5%] vs. 11.5% [95% CI 7.9%–16.7%]).

**Table 4:** Summary rates of the main clinical outcomes among patients with *Streptococcus suis* infection included in a systematic review

Variable	Single-case dataset, n = 174	Large studies, median (range)	Meta-analysis, pooled mean (95% CI)	No. studies meta-analyzed*
Death	10.3	8.9 (0.0–56.0)	12.8 (9.0–18.0)	25
Hearing loss†	44.8	38.7 (6.0–100)	39.1 (31.0–47.8)	26
Recovery from hearing loss	‡	5.0 (0.0–52.3)	15.4 (5.3–37.3)	8
Vestibular dysfunction§	16.7	25.0 (3.3–60.0)	22.7 (15.6–32.0)	13
Visual impairment	4.0	–	–¶	–

*Includes the single-case dataset and the large studies (Technical Appendix Table 2). †Studies included if case-patients were reported to have any degree of hearing impairment (unilateral or bilateral, temporary or permanent). ‡Reliable data could not be extracted for the majority of the case reports. §Studies included if case-patients were reported to have ataxia, vertigo, loss of balance, or vestibular dysfunction. ¶Dashes indicate not applicable because no large study reported these data.

### Clinical Outcomes

Among the survivors, hearing loss (pooled rate 39.1% [95% CI 31.0%–47.8%]) and vestibular dysfunction (22.7% [95% CI 15.6%–32.0%]) were the most common sequelae (Table 4). Reported rates for both sequelae varied widely, even within a country such as Thailand, (Technical Appendix Figures 1–4). Similar to CFRs, there was a marginally positive correlation between hearing loss and meningitis rates (p = 0.05) (Technical Appendix Table 5). The pooled hearing loss rate for studies in which all patients had meningitis was 55.3% (95% CI 36.2%–72.9%), compared with 34.0% (95% CI 26.0%–43.1%) for the remaining studies. For the vestibular dysfunction, none of the investigated study-level factors were significant. Among the Asian countries, the reported rate of vestibular sequelae was lowest in Vietnam (4.0%).

Limited information described how hearing loss and vestibular dysfunction were evaluated during and after infection. Eight of 25 large studies reporting hearing loss indicated whether hearing loss was permanent after hospital discharge. Only 4 described their follow-up processes; follow-up time ranged from 2 months to 30 months (*4*,*8*,*28*,*31*). On the basis of these limited data, we estimated a comparatively low median rate of recovery from hearing loss of 5.0% (range 0%–52.3%) and the pooled rate of 15.4% (95% CI 5.3%–37.3% (Table 4). Hearing loss might be mediated by adjunctive corticosteroid treatments, as was shown in a trial in Vietnam (*34*). Of the *S. suis* patients, 12.3% had deafness in at least 1 ear in the dexamethasone treatment group (n = 57), compared with 37.7% in the placebo group (n = 53).

## Discussion

We have updated estimates of the global prevalence, epidemiology, and clinical characteristics of *S. suis* infections in humans. After possible duplicates were removed, the total number of *S. suis* infections by 2012 was close to 1,600 cases, doubling the figure published in 2009 (*12*). Most of the increase comprised cases from Thailand and Vietnam, placing both countries in the highest disease prevalence stratum in the world. In contrast, only a few cases have been reported from the Americas, particularly the United States, the second largest producer of pigs worldwide (*35*). This low number might be attributable to the high industrialization of pig farming systems in the region. Nevertheless, we saw far more cases in Europe, a region where modern farming operations are presumably similarto those in the Americas. Other plausible explanations include the lower virulence of North American bacterial strains (*36*) or different slaughtering practices.

We counted only published cases; the actual number of cases would be considerably higher, particularly in areas to which *S. suis* is endemic, such as Asian countries with extensive pig rearing. The problem of underestimation is further exacerbated by the fact that *S. suis* infection is not a notifiable disease in many countries. In addition, lack of diagnostic capacities or limited disease awareness in local laboratories can result in undiagnosed or misdiagnosed cases.

Meningitis is the main syndrome in approximately two thirds of *S. suis*–infected patients, although this finding varied by country. The syndromic distribution of the reported cases may depend on study design and case ascertainment methods. All major studies in Vietnam ascertained *S. suis* cases from the population of patients with central nervous system diseases, which could lead to underrepresentation of *S. suis* patients with clinical syndromes other than meningitis. Only 1 patient without meningitis (diagnosed as spontaneous bacterial peritonitis with serotype 16 infection) has been reported in this country (*37*). Nevertheless, whether the existing strains infecting humans in Vietnam mainly cause meningitis remains unclear. In fact, lumbar puncture is performed regularly for all *S. suis*–infected patients, including those with severe sepsis, at a hospital for tropical diseases in Vietnam, and almost all had exhibited typical characteristics of bacterial meningitis in cerebrospinal fluid. On the other hand, meningitis might not be diagnosed or reported from other countries, therefore reducing the global *S. suis* meningitis estimate.

The difference in CFR between case-patients with meningitis and case-patients with severe sepsis has been documented in both outbreak and nonoutbreak situations in China and Thailand (*1*,*2*,*24*). Significantly more deaths were reported among *S. suis* patients with systemic infection, including hypotension, septic shock, multiorgan failure, and disseminated intravascular coagulation in these studies. In the Sichuan outbreak in 2005, the CFR reached 62% for patients classified as having streptococcal toxic shock syndrome (*1*). Several hypotheses have been suggested; however, the pathologic mechanisms underlying this high CFR remain to be elucidated (*7*,*12*). Regarding meningitis cases, the pooled CFR is lower than that for other common causes of adult bacterial meningitis, such as *S. pneumoniae* (19%–37%) (*38*) and *Neisseria meningitidis* (10%) (*39*). However, the rates of sequelae caused by *S. suis* tend to be higher than those caused by other agents reported in a recent meta-analysis (*40*).

We were unable to establish pooled risk estimates for different risk factors because of a lack of studies with appropriate designs. In the Netherlands, the annual risk for *S. suis* meningitis among abattoir workers and pig breeders was 1,500 times higher than that in the general population (*10*). In Vietnam, *S. suis*–infected patients were more likely to have eaten high-risk foods (odds ratio [OR] 4.38), to have pig-related occupations (OR 5.52), and to have pig exposure while having skin injuries (OR 15.96) than community controls (*6*). The lower proportions of patients with occupational exposure in Thailand and Vietnam than in Europe shown in our meta-analysis supports the hypothesis that other risk factors, including food consumption practices, may play a major role in the epidemiology of *S. suis* infection in Asia.

This review is not without limitations. The included studies were highly heterogeneous in quality and in the factors reported, which reduced the number of studies included in each meta-analysis. The summary values of the single-case dataset should be interpreted with caution because the patients in this merged “sample” were heterogeneously “recruited” from different populations, with different assessment protocols. In addition, the studies were mainly retrospective; data could have been easily missed on recall or by re-collecting from the existing data records. We were unable to assess the extent to which this misinformation could affect the overall estimates. However, data collection approach was not significantly associated with the main outcomes examined under this review in our meta-regression analyses.

This review helps to highlight areas in which additional research is needed. Geographic gaps obviously exist in the data on *S. suis* cases, especially in the pig rearing countries in the Americas, Eastern Europe, and Asia, such as Mexico and Brazil, Russia, and the Philippines, respectively. Second, much uncertainty remains in understanding sequelae of *S. suis* infection and recovery from these conditions over time. Careful prospective assessments of these debilitating outcomes and associated social and economic impacts are essential for understanding and reducing the effects of *S. suis* infection. More studies also are needed to assess the treatment effects of adjunctive corticosteroid on hearing loss or other neurologic sequelae.

The effects of *S. suis* infection are mainly in Asia; occupational exposure and eating possibly contaminated foods containing undercooked pig tissues are prime risk factors. Further research in Asia should focus on the factors pertinent to local risk for infection, including the practices of unsafe handling and consumption of pork. Prevention of human infections needs to be tailored for different risk groups, and studies are needed to assess the feasibility and effectiveness of those tailored programs. Additional work is needed to assess the fraction of *S. suis* cases that can be attributed to different risk factors (the population-attributable fraction) and the proportion of *S. suis* cases that might be preventable if specific risk factors were reduced.

Technical Appendix Details of publications in a review of the epidemiology, clinical manifestations, and outcome of *Streptococcus suis* infection in humans.
