# Supplementary material for: Epidemiology, Clinical Manifestations, and Outcomes of Streptococcus suis Infection in Humans
Source: Emerg Infect Dis. 2014 Jul;20(7):1105–14. doi: 10.3201/eid2007.131594 (PMC4073838; doi:10.3201/eid2007.131594)
Supplement: Technical Appendix — Details of publications in a review of the epidemiology, clinical manifestations, and outcome of Streptococcus suis infection in humans. [file 13-1594-Techapp-s1.pdf]

Article DOI: http://dx.doi.org/10.3201/eid2007.131594

Epidemiology, Clinical Manifestations, and Outcome of *Streptococcus suis* Infection

Technical Appendix

Technical Appendix Table 1. Details of 177 included publications in review of the epidemiology, clinical manifestations, and outcome of *Streptococcus suis* infection in humans

PID	Author/year/country/city	Study design	Data collection	Study population	Age group	Recruitment period	*S. suis* diagnosis method	No. *S. suis* cases	Included
7	Agass/1977/England/Oxford	Case report	Retrospective		A	ND	Culture	1	In single dataset
8	Alonso-Socas/2006/Spain/Tenerife	Case report	Retrospective		A		Culture	1	In single dataset
9	Arends/1995/Netherlands/Leiden	Case report	Retrospective		A	1994	Culture	1	In single dataset
11	Arends/1988/Netherlands/Amsterdam	Case series	Retrospective	We only included the Netherlands' cases	A	1968–1984	Culture (CSF)	30	As a large study
12	Asensi/2001/Spain/Oviedo	Case report	Retrospective		A	1998	Culture (blood + CSF)	2	In single dataset
13	Aspiroz/2009/Spain/zaragoza	Case report	Retrospective		A	ND	Culture (blood)	1	In single dataset
14	Atterholm/1985/Sweden/Lund	Case report	Retrospective		A	ND	Culture (CSF + blood)	1	In single dataset
15	Baddeley/1995/UK/Gloucestershire	Case report	Retrospective		A	ND	ND	1	In single dataset
16	Bahloul/2008/France/Salon de Provence	Case report	Retrospective		A	ND	Culture (CSF)	1	In single dataset
24	Berlit/1989/Gemany/Mannheim	Case report	Retrospective		A	ND	Culture (CSF)	1	In single dataset
25	Bezian/1996/France/Bordeaux	Case report	Retrospective		A	ND	Culture (blood + CSF)	1	In single dataset
28	Bouchaud/1997/France/Elbeuf	Case report	Retrospective		A	Feb 1995	Culture (CSF + blood)	1	In single dataset
29	Busova/2010/Czech Republic/	Case report	Retrospective		A	Jul 2005	Culture (CSF)	1	In single dataset
31	Braun/2007/Germany/Augsburg	Case report	Retrospective		A	ND	Culture (CSF + blood)	1	In single dataset
34	Bungener/1989/Federal Republic of Germany	Case report	Retrospective		A	1984	Culture (blood)	1	In single dataset
35	Cammaert/1990/Belgium/	Case report	Retrospective		A	ND	Culture (CSF)	1	In single dataset
36	Camporese/2007/Italy/Pordenone	Case report	Retrospective		A	ND	Culture (CSF)	1	In single dataset
37	Caumont/1996/France/Reims	Case report	Retrospective		A	ND	Culture (disco-vertebral biopsy)	1	In single dataset
38	Chan/2002/Singapore	Cross section	Retrospective	Bacterial meningitis	A	1993–Jun 2000	Culture (blood)	1	In single dataset
39	Chang/2006/Japan	Case series	Retrospective		A	1994–2006	Culture	7	As a large study
41	Chattopadhyay/1980/England/London	Cross section	Combinaton	Meningitis	A	1971–1978	Culture	1	In single dataset
42	Chau/1983/China/Hong Kong	Case series	Retrospective		A	1978–1981	Culture (blood or CSF)	8	As a large study
43	Cheng/1987/China/Hong Kong	Case report	Retrospective		A	ND	Culture (blood + knee aspirate)	1	In single dataset

PID	Author/year/country/city	Study design	Data collection	Study population	Age group	Recruitment period	*S. suis* diagnosis method	No. *S. suis* cases	Included
44	Chotmongkol/1999/Thailand	Case report	Retrospective		A	1997		1	In single dataset
							Culture (blood + CSF)		
47	Clarke/1991/UK/London	Case report	Retrospective		A	ND	Culture (blood + CSF)	1	In single dataset
48	Clements/1982/UK/Cambridge	Case report	Retrospective		A	ND	Culture (1st pt: blood + CSF, 2nd pt: blood)	2	In single dataset
52	Colaert/1985/Belgium	Case report	Retrospective		A	1984	Culture	1	In single dataset
55	Coolen/1989/Belgium	Case report	Retrospective		A	ND	Culture (CSF)	1	In single dataset
56	D'Agostino/2009/Italia/Rome	Cross section	Prospective	Vertebral osteomyelitis		ND	Culture	1	In single dataset
58	De La Hoz/2005/Spain/Cadiz	Case report	Retrospective		A	ND	Culture (blood + CSF)	1	In single dataset
60	Desjars/1987/France/Nantes	Case report	Retrospective		A	Nov 1986	Culture (blood + CSF)	1	In single dataset
61	Dickie/1987/New Zealand	Case report	Retrospective		A	Nov 1986	Culture (blood)	1	In single dataset
62	Donsakul/2003/Thailand/Bangkok	Cross section	Retrospective	Bacterial meningitis	A	1993–1999	Culture	8	As a large study
63	Doube/1988/England/Bath	Case report	Retrospective		A	ND	Culture (synovial pus + blood)	1	In single dataset
64	Dragojlovic/2005/Serbia/Belgrade	Case series	Retrospective		A	2003	Culture (CSF + blood)	5	As a large study
65	Duong/2004/VietNam/HoChiMinh city	Case report	Retrospective		A		Culture (blood, CSF, + lesions fluid)	1	In single dataset
66	Dupas/1992/France/Nantes	Case report	Retrospective		A	Apr 1991	CSF (not specified diagnostic method)	1	In single dataset
67	Durand/2001/France/Grenoble	Case report	Retrospective		A	ND	Culture (CSF)	1	In single dataset
69	Faucqueur/1983/France/Chateaudun	Case report	Retrospective		A	Oct 1981	Culture (CSF, blood)	1	In single dataset
70	Fauveau/2007/France/Blois	Case report	Retrospective		A	ND	Culture (CSF)	1	In single dataset
73	Feng/2009/China/Shenzhen City and Sichuan	Case report	Retrospective		A	Jul–Aug 2007	Culture	3	In single dataset
75	Fernandez/2011/Spain/A Coruna	Case report	Retrospective		A	ND	Culture (CSF + blood)	1	In single dataset
76	Fittipaldi/2009/US/Hawaii	Case report	Retrospective		A		Culture	1	In single dataset
78	Fongcom/2009/Thailand/Lamphun	Case series	Retrospective		A	1999–2000	Culture (blood)	10	As a large study
79	Fongcom/2009/Thailand/Lamphun	Case series	Combine		A	Apr 2001–Apr 2002: Jul 2005–Jul 2007	Culture (blood + CSF)	53	As a large study
80	Francois/1998/France/Limoges	Case report	Retrospective		A	1996	Culture (blood + CSF)	1	In single dataset
81	Galbarro/2009/Spain/Huelva	Case report	Retrospective		A	ND	Culture (CSF)	1	In single dataset
84	Garau/1995/Itali/Cagliari	Case report	Retrospective		A	ND	Culture (CSF)	1	In single dataset
85	Geffner/2001/Spain/Castellon	Case report	Retrospective		A	ND	Culture (blood + CSF)	2	In single dataset
95	Grebe/1997/Germany/Heidelberg	Case report	Retrospective		A	ND	Culture (CSF, blood)	1	In single dataset
97	Halaby/2000/Netherlands/Maastricht	Case report	Retrospective		A	ND	Culture (blood, CSF)	1	In single dataset
98	Haleis/2009/Canada/Manitoba	Case report	Retrospective		A	Oct 2007	Culture (CSF)	1	In single dataset
99	Hantson/1991/Belgium/Brussels	Case report	Retrospective		A	ND	Culture (CSF + blood)	1	In single dataset
100	Hay/1989/UK/Edinnburgh	Case report	Retrospective		A	Apr–1987	Culture (CSF)	2	In single dataset
102	Heidt/2005/Germany/Giessen	Case report	Retrospective		A		Culture (blood + aortic valve)	1	In single dataset
103	Helbok/2006/Thailand/Bangkok	Cross section	Retrospective	Chronic meningitis		1993–1999	Culture	2	In single dataset
105	Hidalgo/2007/Spain/Malaga	Case report	Retrospective		A		Culture (CSF + blood)	1	In single dataset

PID	Author/year/country/city	Study design	Data collection	Study population	Age group	Recruitment period	*S. suis* diagnosis method	No. *S. suis* cases	Included
106	Ho/1990/China/Hong Kong	Case report	Retrospective		A	Aug 1989	Culture (blood)	1	In single dataset
108	Hoa/1998/Vietnam/Ho Chi Minh	Cross section	Prospective	Community acquired septicemia		1 Jun 1993–30 May 1994	Culture (blood)	4	In single dataset
110	Hu/2000/China/Jiangsu	Outbreak investigation	Retrospective			Jul 1998	Culture (CSF or blood)	25	As a large study
112	Huang/2005/China/Taiwan/Taipei	Case report	Retrospective		A	Mar 2000, Sep 2002	Culture (CSF, blood)	2	In single dataset
113	Huh/2011/Korea/Seoul	Case report	Retrospective		A		Culture (CSF + blood)	1	In single dataset
114	Hui/2005/China/Hongkong	Cross section	Combine	Bacterial meningitis	A	1992–2001	Culture	6	As a large study
115	Ibaraki/2003/Japan/Nagaoka	Case report	Retrospective		A	Aug 2002	Culture (CSF)	1	In single dataset
116	Ip/2007/Hongkong	Case series	Retrospective		8A, 2ND	1995–2005	Culture (CSF or blood)	10	As a large study
121	Juncal/1997/Spain/La Coruna	Case report	Retrospective		A	ND	Culture (CSF + blood)	1	In single dataset
122	Kaufhold/1988/Germany/Koln	Case report	Retrospective		A	Sep 1987	Culture (CSF + blood)	1	In single dataset
125	Kay/1995/China/Hong Kong	Case series	Retrospective		A	1984–1993	Culture (blood and/or CSF)	25	As a large study
126	Kennedy/2008/Australia/New South Wales	Case report	Retrospective		A	Oct 2006, Jan 2008	Culture (blood)	2	In single dataset
127	Kerdsin/2009/Thailand	Cross section	Retrospective	Unidentified streptoccocal isolates	A	Jan 2006–Sep 2008	Culture (blood or CFS)	165	As a large study
128	Kerdsin/2011/Thailand/Uttaradit	Case report	Retrospective		A	Jun and May 2007	Culture (1 case acitic fluid, 1 case blood)	2	In single dataset
129	Kerdsin/2009/Thailand	Cross section	Retrospective	Confirmed *S. suis*	A	Jan 2006–Sep 2008	Culture (blood + CSF)	12	As a large study
130	Khin Thi/1985/China/HongKong	Case series	Prospective		A	1982–1984	Culture (CSF, blood)	30	As a large study
131	Kim/2011/Korea/Chungcheong	Case report	Retrospective		A	ND	Culture (blood, joint fluid)	1	In single dataset
134	Kohler/1989/Germany/	Case report	Retrospective		A	Jun 1987	Culture (CSF+ blood)	1	In single dataset
135	Kopic/2002/Croatia/Slavonski Broad	Case report	Retrospective		A	Nov 2000	Culture (1 blood, 1 CFS)	2	In single dataset
137	Kowalik/2007/Poland/Gdansk	Cross section	Prospective	Community acquired bacterial meningitis	A	Jan 1999–Dec 2001	Culture (CSF)	3	In single dataset
138	Laohapensang/2010/Thailand/Chiang Mai	Case report	Retrospective		A	ND	Culture (blood, aspirated synovial fluid)	1	In single dataset
139	Lecuyer/2004/France/Saint Quentin	Case report	Retrospective		A	ND	ND	1	In single dataset
140	Lee/2008/US/San Francisco	Case report	Retrospective		A	2003	Culture (blood)	1	In single dataset
141	Leelarasamee/1997/Thailand/bangkok	Case report	Retrospective		A	ND	Culture (blood + CSF)	3	In single dataset
145	Lopreto/2005/Agentina/La Plata	Case report	Retrospective		A	ND	Culture (CSF)	1	In single dataset
146	Luengo/2006/Spain/Caceres	Case report	Retrospective		A	2004	Culture (CSF+ blood)	1	In single dataset
149	Lutticken/1986/Germany/Munich	Case report	Retrospective		A	1985	Culture (CSF, blood)	1	In single dataset
150	Ma/2008/China/HongKong	Case series	Retrospective		A	1 Jan 2003–31 Jul 2005	Culture (CSF, blood)	21	As a large study
152	Maher/1991/UK/Leeds	Case report	Retrospective		A	ND	Culture (blood)	1	In single dataset
153	Mai/2008/VietNam/HCMC	Cross section	Prospective	Bacterial meningitis	A	Nov 1996–Jun 2005	Culture + PCR	151	As a large study

PID	Author/year/country/city	Study design	Data collection	Study population	Age group	Recruitment period	*S. suis* diagnosis method	No. *S. suis* cases	Included
155	Manzin/2008/Italy/Sardinia	Case report	Retrospective		A	Nov 2007	Culture (CSF)	1	In single dataset
156	Martimez Aviles/1994/Spain/Madrid	Case report	Retrospective		A	ND	Culture (blood)	1	In single dataset
157	Matano/1984/Italy/Caserta	Case report	Retrospective		A	Jan 1983	Culture (CSF)	1	In single dataset
158	Matsuo/2003/Japan/Nagasaki	Case report	Retrospective		A	Feb 2002	Culture (CSF)	1	In single dataset
159	Mazokopakis/2005/Greece/Crete	Case report	Retrospective		A	ND	Culture (blood, CSF)	1	In single dataset
160	McLedon/1978/Leicester/UK	Case report	Retrospective		A	1976	Culture (CSF, blood)	1	In single dataset
161	McNeil/1986/London/UK	Case report	Retrospective		A	ND	Culture (blood)	1	In single dataset
162	Meecham/1992/England/Chester	Case report	Retrospective		A	ND	Culture (blood, CSF)	1	In single dataset
163	Michaud/1996/Canada/Quebec	Case report	Retrospective		A	1994	Culture (CSF)	1	In single dataset
165	Nagel/2008/Agentina/Santa Fe	Case report	Retrospective		A	ND	Culture (blood + CSF)	1	In single dataset
166	Navacharoen/2009/Thailand/Northern	Case series	Retrospective		A	Jan 2003–Jan 2007	Culture	40	As a large study
167	Nghia/2008/VietNam/Long An	Case report	Retrospective		A	2001	Culture (blood)	1	In single dataset
168	Nghia/2011/VietNam/Ho Chi Minh city	Case control	Prospective	Hospital cases and controls, community controls	A	May 2006–Jun 2009	Culture (blood, CSF)	101	As a large study
172	Pedroli/2003/France/Montbrison	Case report	Retrospective		A	Nov 2000	Culture (blood)	1	In single dataset
173	Peetermans/1989/Netherlands/Leiden	Case report	Retrospective		A	1988	Culture (blood)	1	In single dataset
174	Perseghin/1995/Italy/Sondalo	Case report	Retrospective		A	ND	Culture (CSF)	1	In single dataset
175	Piech/2009/France/Clermont-Ferrand	Case report	Retrospective		A	ND	Culture (blood)	1	In single dataset
177	Poggenborg/2008/Denmark/Copenhag en	Case report	Retrospective		A	2006	Culture (CSF, blood)	1	In single dataset
179	Pychova./2011Czech/Brno	Case report	Retrospective		A	2008; 2009	CSF culture and PCR	2	In single dataset
180	Plotek/2007/Poland/Kalisz	Case report	Retrospective		A		Culture (blood + CSF)	1	In single dataset
183	Rao/2008/UK/Kent	Case report	Retrospective		A	ND	Culture (CSF)	1	In single dataset
186	Riquelme/2008/Spain/Santander	Case report	Retrospective		A		Culture (CSF + blood)	1	In single dataset
190	Rosenkranz/2003/Germany	Case report	Retrospective		A	ND	Culture (blood, CSF)	1	In single dataset
191	Rosenstingl/2008/France/Coulommier s	Case report	Retrospective		A	ND	Culture (blood)	1	In single dataset
192	Rusmeechan/2008/Thailand/Phitsanul ok	Case series	Retrospective		A	2001–2006	Culture (blood, CSF)	41	As a large study
197	Seidel/1995/Germany/Munster	Case report	Retrospective		A	Jan 1994	Culture (CSF)	1	In single dataset
198	Shneerson/1980/UK/London	Case report	Retrospective		A	ND	Culture (blood + CSF)	1	In single dataset
199	Sia/2006/China/Hong Kong	Case report	Retrospective		A	2004	Culture (blood, CSF)	1	In single dataset
202	Soemirien/2010/Netherlands/Amsterda m	Case series	Prospective	Adult community-acquired bacterial meningitis	A	Mar 2006–Jan 2009	Culture (CSF)	2	In single dataset
204	Spiss/1999/Austria/Innsbruck	Case report	Retrospective		A	ND	Culture (CSF)	1	In single dataset
206	Strangmann/2002/Germany/Oldenber g	Case report	Retrospective		A	ND	Culture (blood)	1	In single dataset
208	Suankratay/2004/Thailand/Bangkok	Case series	Retrospective		A	Jan 1997–May 2002	Culture (blood + CSF)	12	As a large study
209	Taipa/2008/Portugal?Porto	Case report	Retrospective		A	ND	Culture (CSF)	1	In single dataset

PID	Author/year/country/city	Study design	Data collection	Study population	Age group	Recruitment period	*S. suis* diagnosis method	No. *S. suis* cases	Included
212	Tambyah/1997/Singapore	Case report	Retrospective		A	ND	Culture (blood)	1	In single dataset
213	Tan/2010/Singapore/	Case report	Retrospective		A	ND	Culture (blood)	1	In single dataset
214	Tang/2006/China/Sichuan	Outbreak investigation	Retrospective		A	Jun 2005	Culture + PCR	204	As a large study
216	Taradas/2001/Spain/Castellon	Case report	Retrospective		A	ND	culture (CSF)	2	In single dataset
217	Tayoro/1996/France/Tours	Case report	Retrospective		A	1994	Culture (blood)	1	In single dataset
219	Thwaites/2002/Viet Nam/Ho Chi Minh city	Cross section	Prospective	Bacterial meningitis of tuberculous meningitis	A	1997–2000	Culture (CSF)	31	As a large study
222	Tramontana/2008/Australia/Melbourne	Case report	Retrospective		A	2007	Culture (blood)	1	In single dataset
224	Tran/2011/Viet Nam/HCMC	Cross section	Prospective	Baterial meningitis	A	May 2006–Jun 2009	Culture (blood, CSF)	110	As a large study
225	Trottier/1991/Canada/Quebec	Case report	Retrospective		A	ND	Culture (blood)	1	In single dataset
226	Tsai/2005/China/Taiwan	Case report	Retrospective		A	ND	Culture (CSF)	1	In single dataset
227	Twort/1981/England/Chichester	Case report	Retrospective		A	ND	Culture (CSF + blood)	1	In single dataset
230	van de Beek/2008/Netherlands/nationwide	Cross section	Prospective	Community-acquired bacterial meningitis	A	Oct 1998–Apr 2002	Culture (CSF)	4	In single dataset
231	van Jaarsveld/1990/Netherlands/Nijimegen	Case report	Retrospective		A	April 1989	Culture (blood)	1	In single dataset
232	Van Ooteghem/1988/Belgium/Gent	Case report	Retrospective		A	Mar1986	Culture (blood + CSF)	1	In single dataset
235	Vilaichone/2002/Thailand/Bangkok	Case series	Retrospective			1994–2001	Culture (blood or CSF or peritoneal fluid)	17	As a large study
236	Voutsadakis/2006/Greece/Larissa	Case report	Retrospective		A	ND	Culture (blood)	1	In single dataset
237	Walsh/1992/UK/	Case series	Retrospective		A	1975–1990	Culture (blood or CSF)	35	As a large study
240	Wangkaew/2006/Thailand/ChiangMai	Case series	Retrospective		A	May 2000–Dec 2002	Culture	41	As a large study
241	Wangsomboonsiri/2008/Thailand/Nokornsawan	Case series	Retrospective		A	Jan 2005–Oct 2007	Culture	66	As a large study
242	Watkins/2001/UK/Leeds	Case report	Retrospective		A	Aug 1999	Culture (blood)	1	In single dataset
244	Wertheim/2009/Viet Nam/Ha Noi	Case series	Retrospective		A	Jan–Dec 2007	Culture, PCR	50	As a large study
245	Wilenburg/2006/US/	Case report	Retrospective		A	ND	Culture (blood + CSF)	1	In single dataset
248	Woo/1987/China/Hongkong	Case report	Retrospective		A	ND	Culture (blood + CSF)	2	In single dataset
252	Yamaguchi/1994/Japan	Case report	Retrospective		A	ND	Culture (blood)	1	In single dataset
253	Yang/2005/China/Taiwan	Case report	Retrospective		A	ND	Culture (CSF)	1	In single dataset
254	Yang/2009/China	Case report	Retrospective		A	Jul 2005		4	In single dataset
257	Ye/2009/China/	Outbreak investigation	Retrospective		A	2005	Culture	38	As a large study
259	Yen/1994/China/Taiwan/Kaohsiung	Case report	Retrospective		A	Apr1991	Culture (CSF)	1	In single dataset
261	Yu/2006/China/Sichuan	Outbreak investigation	Retrospective		A	Jun–Aug 2005	Culture	215	As a large study
269	Chau/1983/China/Hong Kong	Case report	Retrospective		A	ND	Culture (blood)	1	In single dataset
270	Koldkjaer/1972/Danmark/Odense	Case report	Retrospective		A	ND	Culture (CSF + blood)	1	In single dataset
271	Perch/1968/Denmrk/Hillerød	Case report	Retrospective		A	1960–1966	Culture (blood + CSF)	3	In single dataset

PID	Author/year/country/city	Study design	Data collection	Study population	Age group	Recruitment period	*S. suis* diagnosis method	No. *S. suis* cases	Included
272	Hickling/1976/England/London	Case report	Retrospective		A	ND	Culture (CSF + blood)	1	In single dataset
273	Kloppenburg/1975/Netherlands/Groningen	Case report	Retrospective		A	ND	Culture (blood)	1	In single dataset
281	Khadthasrima/2008/Thailand/Phayao	Outbreak investigation	Retrospective		A	12 April–11 May/2007	Culture (CSF + blood)	50	As a large study
282	Joynson/1980/Wales/West Glamorgan	Case report	Retrospective		A	ND	Culture (blood + CSF)	1	In single dataset
283	Peel/1979/UK/York	Case report	Retrospective		A	ND	Culture (blood)	1	In single dataset
290	Damodaram/2011/India/Andhra Pradesh	Cross section	Retrospective	Myositis		2006–2011		1	In single dataset
295	Isaradisaikul/2010/Thailand	Cross section	Retrospective	Vertigo	A	April 2005–December 2007	ND	1	In single dataset
296	Bronstein/1995/UK/London	Case report	Retrospective			ND	ND	1	In single dataset
297	Pachirat/2012/Thailand	Cross section	Prospective	Infective endocarditis	A	January 2010–December 2011	PCR, culture (blood)	3	In single dataset
298	Takeuchi/2012/Thailand/Phayao	Cross section	Prospective		A	2010	Culture (blood or CSF)	31	As a large study
299	Moon/2011/Korea	Case report	Retrospective		A		Culture (blood, joint fluid)	1	In single dataset
300	Nghia/2012/Vietnam	Cross section	Prospective	CNS infections	A	August 2007–April 2010	Culture (blood, CSF) + PCR	147	As a large study
302	Boonyagars/2010/Thailand/	Case report	Retrospective		A	ND	Culture (blood + CSF)	1	In single dataset
303	Choi/2012/Korea/Gwangju	Case report	Retrospective		A	ND	Culture (CSF)	1	In single dataset
304	Tsai/2012/China/Taiwan/	Case series	Retrospective		A	2000, 2002, 2005, 2007, 2009, 2010, 2010, 2011	PCR	8	As a large study
305	Thayawiwat/2012/Thailand/Phayao	Cross section	Prospective	Sepsis, meningitis, IE, pneumonia, arthritis	A	January 2009–December 2011	Culture (blood or CSF)	31	As a large study
307	Taylor/2012/Vietnam/Hanoi	Cross section	Prospective	CNS infections	A	May 2007–December 2008	Culture (blood or CSF) + PCR	48	As a large study
308	Hanterdsith/2012/Thailand/	Case report	Retrospective		A		Culture (blood_	1	In single dataset
309	Paul/1977/France/	Case report	Retrospective		A		ND	1	In single dataset
310	Avril/1977/France/Paris	Case report	Retrospective		A		Culture (blood + CSF)	2	In single dataset
311	Perch/1971/Denmark/Copenhagen	Case report	Retrospective		A		Culture (CSF)	1	In single dataset
312	Yoon/2012/Korea/Jeju	Case report	Retrospective		A	ND	Culture (blood + pleural fluid)	1	In single dataset
313	de Ceuster/2012/Netherlands	Case report	Retrospective		A	ND	Culture (blood + CSF)	1	In single dataset
314	Zalas-Wiêcek/2012/Poland/Bydgoszcz	Case report	Retrospective		A	ND	Culture (blood + CSF)	1	In single dataset
315	Vela/2012/Spain/	Case report	Retrospective		A	ND	Culture (blood)	1	In single dataset

*PID, publication identification ; A, adult; ND, not determined

Technical Appendix Table 2. Articles included in the meta-analysis of each variable

Variable	No. studies included	PID of papers included
Mean age, y	25	Pooled single-case dataset, and papers PID 261, 127, 153, 224, 241, 79, 244, 281, 192, 240, 166, 237, 298, 11, 130, 125, 235, 129, 208, 116, 42, 62, 304, 39.
Male sex	26	Pooled single-case dataset, and papers PID 261, 127, 153, 224, 241, 79, 244, 281, 192, 240, 166, 237, 298, 305, 11, 130, 125, 150, 235, 208, 42, 62, 304, 39, 64.
Pig-related occupation	21	Pooled single-case dataset, and papers PID 153, 168, 241, 240, 166, 237, 298, 11, 261, 42, 150, 114, 208, 235, 62, 125, 304, 129, 110, 39.
History of contact with pig/pork	14	Pooled single-case dataset, and papers PID 168, 240, 261, 150, 208, 235, 127, 224, 244, 130, 110, 39, 64.
History of eating high-risk pork food	9	Pooled single-case dataset, and papers PID 168, 241, 166, 298, 235, 127, 79, 244.
Skin injury	8	Pooled single-case dataset, and papers PID 168, 237, 261, 150, 235, 125, 39.
Drinking history	13	Pooled single-case dataset, and papers PID 127, 168, 241, 79, 244, 240, 166, 305, 125, 150, 235, 208.
Concurrent diabetes	9	Pooled single-case dataset, and papers PID 224, 241, 240, 192, 166, 298, 125, 150.
Meningitis	26	Pooled single-case dataset, and papers PID 261, 127, 153, 300, 224, 241, 79, 244, 192, 240, 166, 237, 298, 130, 11, 125, 110, 150, 235, 129, 208, 304, 42, 62, 64.
Sepsis	12	Pooled single-case dataset, and papers PID 261, 127, 241, 79, 240, 166, 298, 130, 150, 235, 129.
Endocarditis	10	Pooled single-case dataset, and papers PID 127, 241, 79, 240, 166, 125, 150, 235, 62.
Endophthalmitis	9	Pooled single-case dataset, and papers PID 241, 244, 240, 166, 237, 130, 39, 64.
Arthritis	12	Pooled single-case dataset, and papers PID 127, 241, 237, 298, 130, 125, 150, 208, 42, 62, 39.
Spondylodiscitis	3	Pooled single-case dataset, and papers PID 241, 79, 240
Toxic shock syndrome	4	Pooled single-case dataset, and papers PID 110, 79, 214
Mean onset–admission duration	7	Pooled-single case dataset, and papers PID 241, 244, 240, 298, 208, 150.
Mean hospitalization duration	5	Pooled single-case dataset, and papers PID 241, 244, 208, 129.
Meningeal sign	18	Pooled single-case dataset, and papers PID 127, 153, 224, 79, 244, 240, 192, 166, 257, 237, 125, 150, 235, 208, 62, 304, 39.
Skin rash	10	Pooled single-case dataset, and papers PID 214, 153, 79, 244, 192, 11, 130, 110, 62.
Shock	12	Pooled single-case dataset, and papers PID 127, 153, 241, 79, 244, 240, 11, 125, 110, 150, 235.
Respiratory failure	6	Pooled single-case dataset, and papers PID 214, 79, 244, 129, 64.
Acute renal failure	5	Pooled single case dataset, and papers PID 153, 244, 240, 235.
Disseminated intravascular coagulation	9	Pooled single case dataset, and papers PID 214, 241, 240, 130, 125, 150, 235, 39.
Relapse	5	Pooled single-case dataset, and papers PID 11, 237, 125, 208.
Death	25	Pooled single-case dataset, and papers PID 39, 241, 79, 281, 244, 240, 192, 166, 237, 298, 130, 11, 125, 110, 261, 150, 235, 127, 153, 208, 224, 62, 304, 64.
Hearing loss	26	Pooled single-case dataset, and papers PID 39, 241, 79, 281, 244, 240, 192, 166, 237, 298, 130, 11, 125, 150, 235, 127, 153, 208, 224, 42, 62, 257, 305, 304, 64.
Recovery from hearing loss	8	Papers PID 79, 192, 166, 237, 125, 235, 153, 62.
Vestibular dysfunction	13	Pooled single-case dataset, and papers PID 79, 244, 192, 166, 237, 11, 125, 208, 42, 62, 304, 64.
Blood leukocytes, 109/L	9	Pooled single-case dataset, and papers PID 261, 153, 241, 244, 240, 125, 235, 208.
Blood platelet, 109/L	7	Pooled single-case dataset, and papers PID 261, 153, 241, 244, 240, 208.
CSF leukocytes/mm3	7	Pooled single-case dataset, and papers PID 153, 241, 244, 125, 208, 42.
CSF protein, g/L	7	Pooled single-case dataset, and papers PID 153, 241, 244, 125, 208, 42.
CSF glucose, mg/dL	6	Pooled single-case dataset, and papers PID 241, 244, 125, 208, 42.

*PID, Publication Identification; CSF, cerebrospinal fluid.

Technical Appendix Table 3. Summary of preexisting conditions (other than diabetes) of human *Streptococcus suis* cases worldwide

Preexisting condition	No. cases	Publication ID
Heart disease	14 (10 had infective endocarditis as the main syndrome)	240, 62, 208, 235, 106, 213, 217
Hypertension	13	213, 166, 298, 302, 159, 121, 8, 113, 179, 195
Cirrhosis	10	166, 298, 128, 167, 150
Cancer	5	240, 208, 150, 236, 155
Dyslipidemia/hyperlipidemia	2	166, 302
Tuberculosis	2	150, 125
Psoriasis	2	150
Chronic liver disease	1	79
Aplastic anemia	1	298
Spinal canal stenosis	1	298
Rheumatoid arthritis	1	298
Chronic pancreatitis	1	84
Pelger-Huet anomaly	1	135
Splenectomy	9	168, 135, 15, 180, 242, 58, 80, 145, 237
History of corticoid use	2	208
Past *S. suis* infection	1	80

Technical Appendix Table 4. Microbiological characteristics of the merged single-case dataset in a systematic review of *Streptococcus suis* infection*

Characteristic	No. (%)
Microbiological diagnosis, n = 141	
Blood culture only	33 (23.4)
CSF culture only	34 (24.1)
Both CSF and blood culture, no PCR	53 (37.6)
PCR and culture (blood and/or CSF)	18 (12.8)
PCR only	3 (2.1)
Initial misdiagnosis, n = 174 (9 viridans streptococci, 2 *S. faecalis*, 1 *S. acidominimus* and 1 *S. bovis*)	13 (7.5)
Serotype 2, n = 174	94 (54)
Penicillin sensitivity, n = 92	90 (97.8)
Penicillin resistance, n = 92	1 (1.1)

*CSF, cerebrospinal fluid.

Technical Appendix Table 5. Bivariate meta-regression for meningitis, death, and hearing loss among included studies in the systematic review of *Streptococcus suis* infection*

			Meta-regression†	
Study-level factor	No. studies	Pooled group event rate (95% CI)	Rate difference	p value
**Meningitis**				
Country of publication				
Other‡[†]	4	0.731 (0.381–0.923)	–0.258	0.773
China mainland	2	0.456 (0.375–0.540)	–0.538	<0.001
Hong Kong	4	0.789 (0.526–0.927)	–0.226	1.000
Thailand	11	0.545 (0.434–0.651)	–0.436	<0.001
Vietnam	4	0.995 (0.981–0.999)	reference	
Year of publication				
2005 and earlier	10	0.771 (0.599–0.884)	0.034	0.824
After 2005	15	0.633 (0.513–0.738)	reference	
Study design				
Case series	17	0.670 (0.539–0.779)	–0.234	0.054
Outbreak	2	0.456 (0.375–0.540)	–0.404	0.004
Cross-sectional	6	0.920 (0.744–0.978)	reference	
Data collection				
Retrospective	19	0.595 (0.504–0.680)	–0.298	0.003
Prospective	6	0.931 (0.6960.988)	reference	
**Death**				
Country of publication				
Other‡	5	0.100 (0.051–0.188)	0.063	1.000
China mainland	2	0.338 (0.085–0.739)	0.198	0.060
Hong Kong	3	0.099 (0.025–0.320)	0.096	1.000
Thailand	11	0.158 (0.098–0.244)	0.129	0.264
Vietnam	3	0.030 (0.011–0.080)	reference	
Year of publication				
2005 and earlier	9	0.163 (0.079–0.306)	0.069	0.316
After 2005	15	0.112 (0.071–0.173)	reference	
Study design				
Case series	16	0.147 (0.097–0.216)	0.103	0.159
Outbreak	3	0.216 (0.064–0.525)	0.140	0.070
Cross-sectional	5	0.059 (0.024–0.140)	reference	
Data collection				

Retrospective	19	0.135 (0.093–0.190)	0.035	0.522
Prospective	5	0.108 (0.030–0.322)	Reference	
Meningitis rate				
<0.5	7	0.244 (0.143–0.384)	0.203	0.001
0.5– 0.9	8	0.149 (0.104–0.210)	0.106	0.106
>0.9	7	0.040 (0.022–0.070)	Reference	
**Hearing loss**				
Country of publication				
Other‡	5	0.479 (0.344–0.619)	–0.014	1.000
China mainland	1	0.237 (0.128–0.396)	–0.245	1.000
Hong Kong	4	0.520 (0.278–0.753)	0.018	1.000
Thailand	12	0.313 (0.208–0.442)	–0.170	1.000
Vietnam	3	0.437 (0.205–0.699)	Reference	
Year of publication				
2005 and earlier	9	0.508 (0.388–0.627)	0.128	0.327
After 2005	16	0.331 (0.231–0.450)	Reference	
Study design				
Case series	17	0.419 (0.311–0.536)	0.012	1.000
Outbreak	2	0.131 (0.031–0.413)	–0.266	0.420
Cross-sectional	6	0.422 (0.248–0.617)	Reference	
Data collection				
Retrospective	19	0.385 (0.285–0.497)	–0.096	0.327
Prospective	6	0.399 (0.238–0.586)	Reference	
Meningitis rate				
<0.5	5	0.236 (0.113–0.428)	–0.303	0.066
0.5–0.9	9	0.356 (0.259–0.466)	–0.241	0.051
>0.9	8	0.586 (0.400–0.751)	Reference	

*Boldface indicates dependent variable being analyzed in meta-regression.
†General linear model, weighted by sample size, study-level factors were included as random factors, with adjustment for multiple comparisons by the Bonferroni method.
‡Includes the United Kingdom, the Netherlands, Serbia, Taiwan, and Japan.

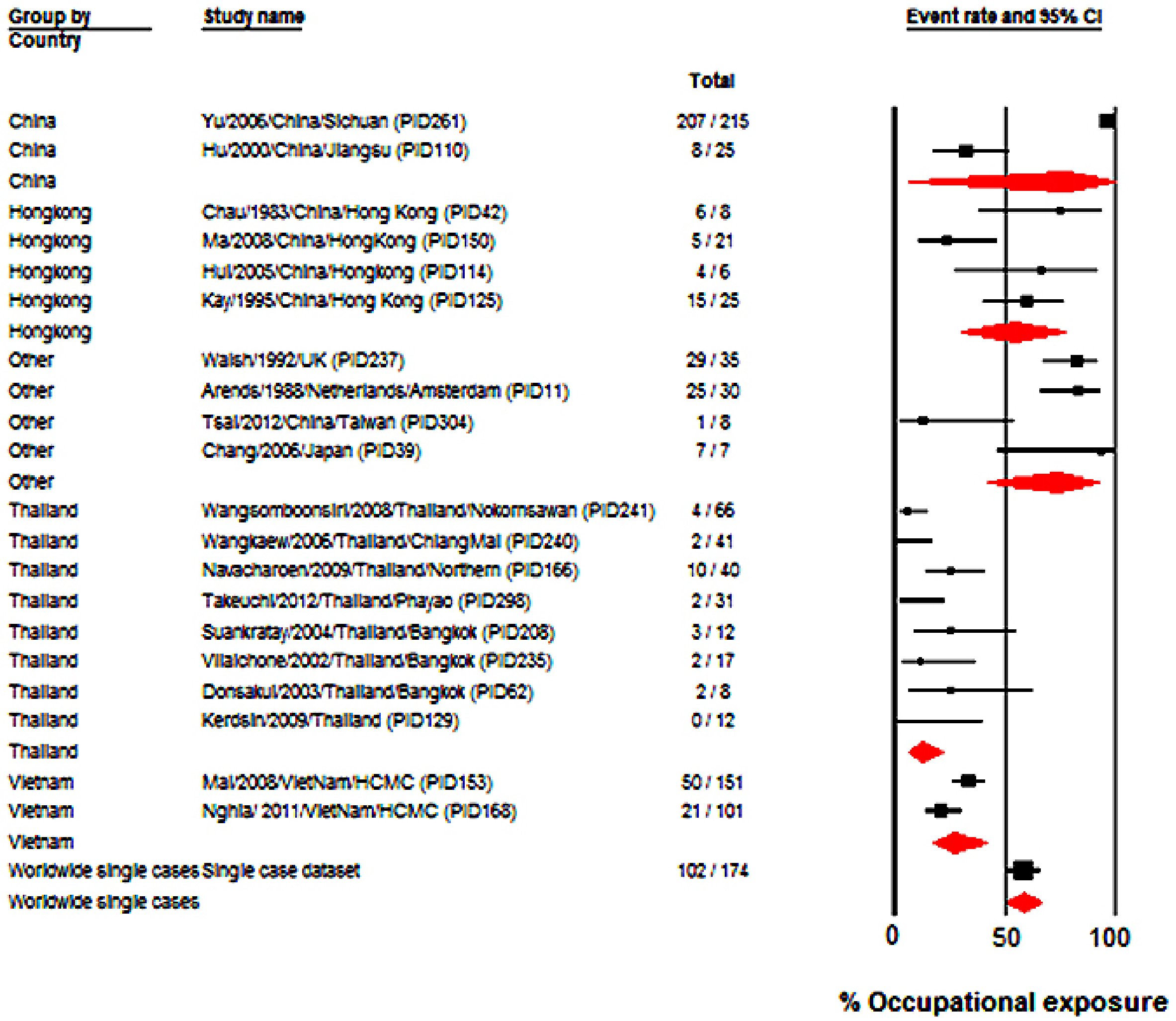

Figure 1. Forest plot of subgroup meta-analysis (random-effects) for proportions of *S. suis* patients with pig-related occupation by country reported in the 21 included studies. For each study, proportion and 95% CI is presented, with the size proportional to the study weight. The red rhombus presents the pooled proportion for each country group.

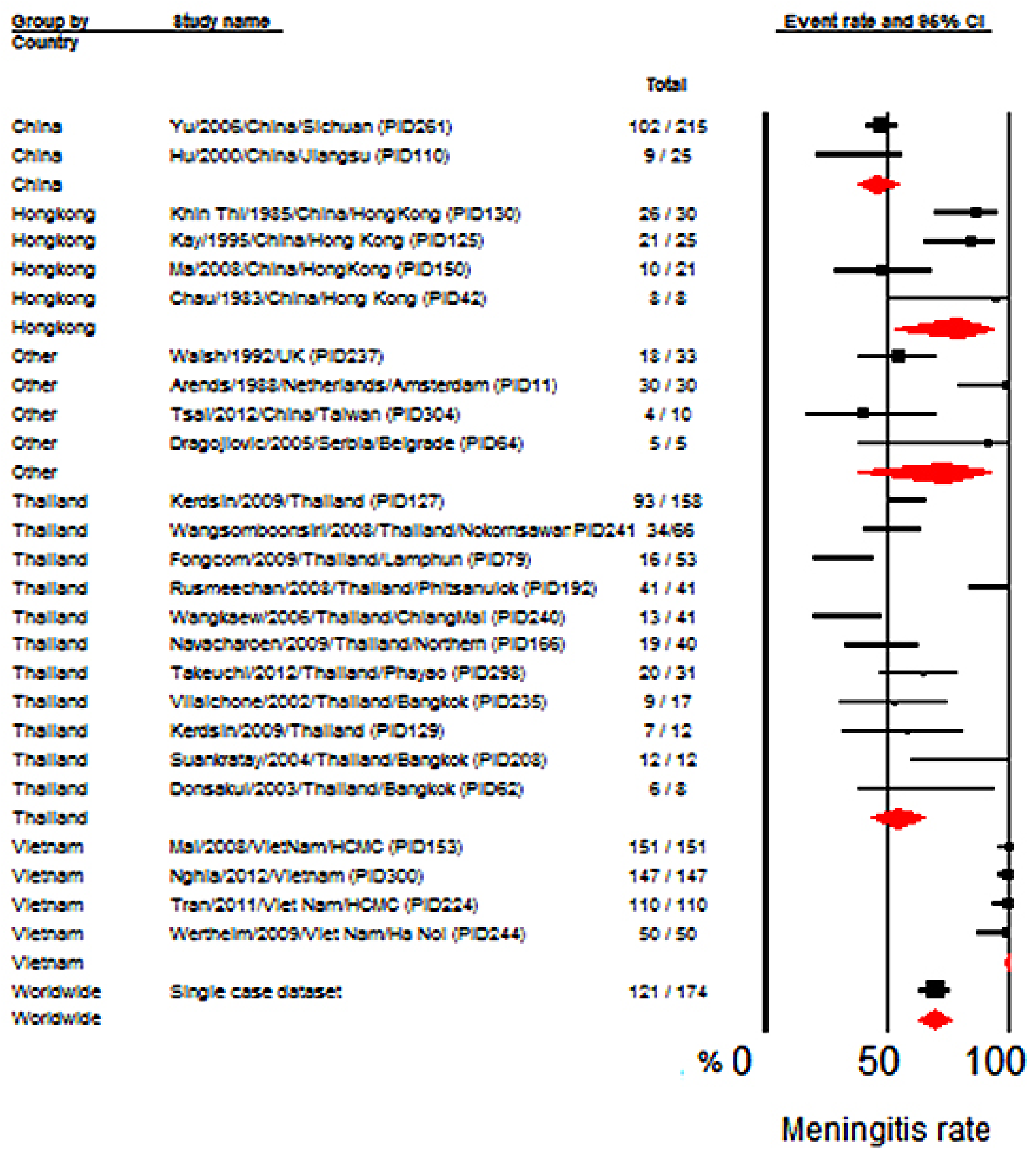

Figure 2. Forest plot of subgroup meta-analysis (random-effects) for the rates of meningitis by country reported in the 26 included studies. For each study, the event rate of meningitis and 95% CI is presented; size is proportional to study weight. The red rhombus presents the pooled event rate for each country group.

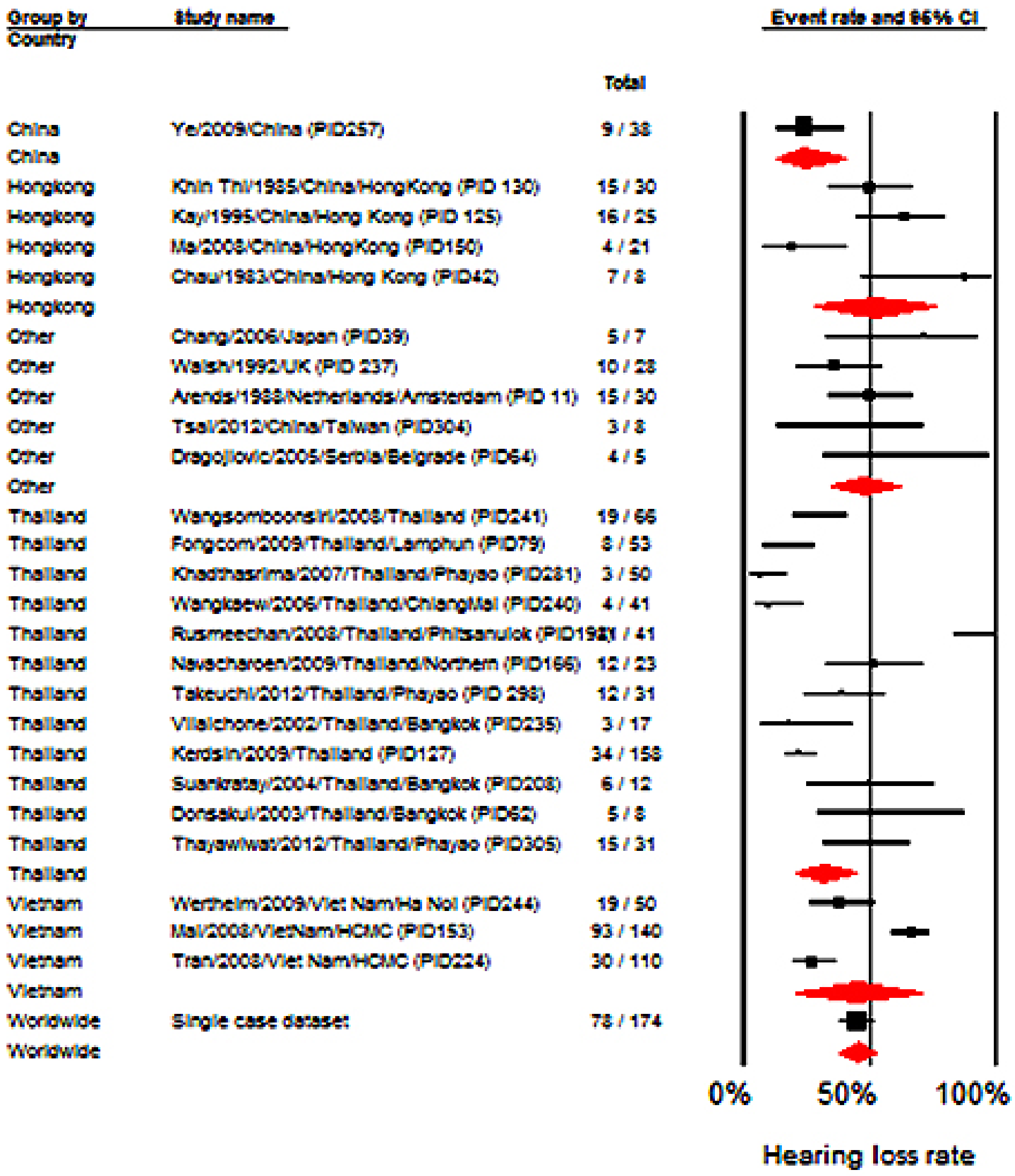

Figure 3. Forest plot of subgroup meta-analysis (random-effects) for the rates of hearing loss reported in the 26 included studies by country. For each study, the event rate and 95%CI is presented; size proportional to study weight. The red rhombus presents the pooled event rate for each country group.

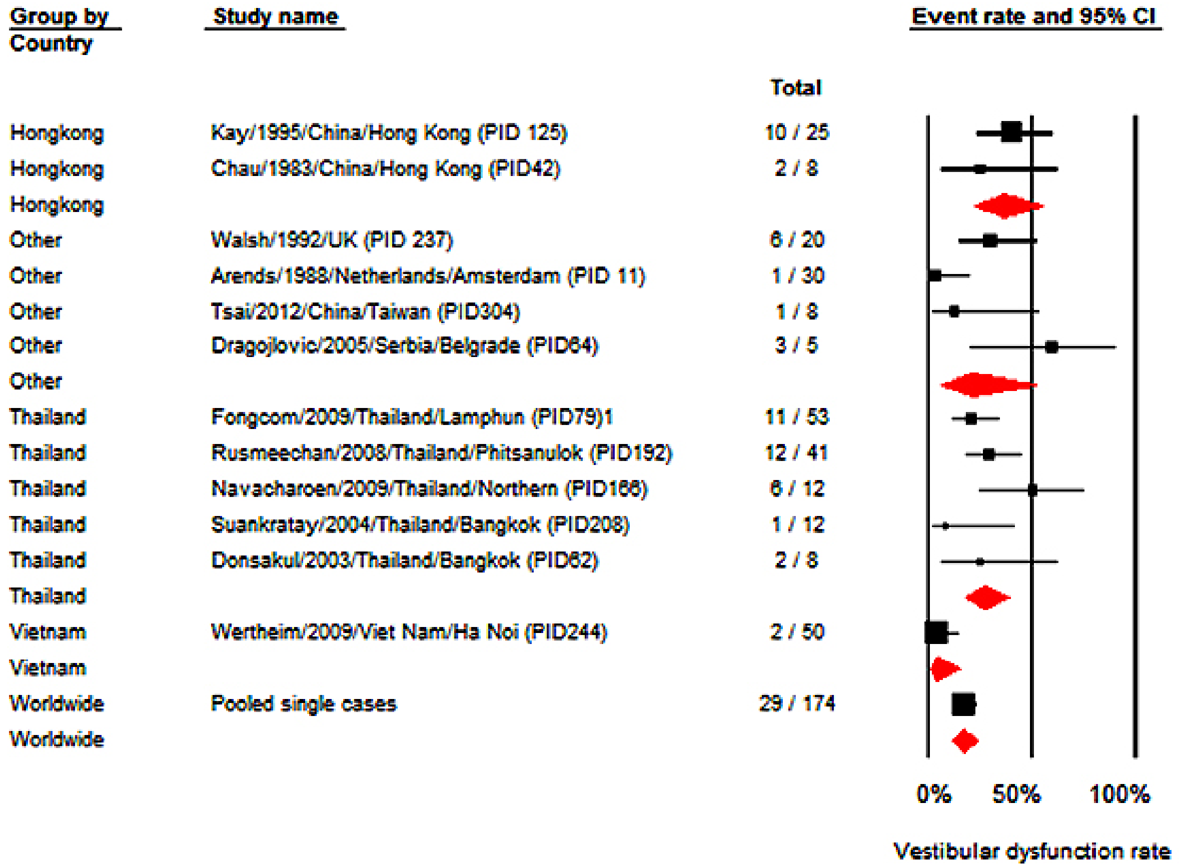

Figure 4. Forest plot of subgroup meta-analysis (random-effects) for the rates of vestibular dysfunction by country reported in the 13 included studies. For each study, the event rate and 95% CI is presented; size is proportional to study weight. The red rhombus presents the pooled event rate for each country group.
